# Nimodipine improves cortical efficiency during working memory in healthy subjects

**DOI:** 10.1038/s41398-020-01066-z

**Published:** 2020-11-02

**Authors:** Caroline F. Zink, Mellissa Giegerich, Greer E. Prettyman, Kayla E. Carta, Marcus van Ginkel, Molly P. O’Rourke, Eesha Singh, Edward J. Fuchs, Craig W. Hendrix, Eric Zimmerman, Jennifer Breakey, Mark A. Marzinke, Pamela Hummert, Jay J. Pillai, Daniel R. Weinberger, Kristin L. Bigos

**Affiliations:** 1 Baltimore Research and Education Foundation, Baltimore, MD United States; 2 Lieber Institute for Brain Development, Baltimore, MD United States; 3 Department of Psychiatry and Behavioral Sciences, Johns Hopkins School of Medicine, Baltimore, MD United States; 4 Veterans Administration, San Diego, CA United States; 5 Department of Neuroscience, University of Pennsylvania, Philadelphia, PA United States; 6 Department of Medicine, Division of Clinical Pharmacology, Johns Hopkins School of Medicine, Baltimore, MD United States; 7 School of Nursing, University of Pennsylvania, Philadelphia, PA United States; 8 College of Medicine, University of Tennessee, Memphis, TN United States; 9 Department of Pharmacology and Molecular Science, Johns Hopkins School of Medicine, Baltimore, MD United States; 10 Department of Epidemiology, Johns Hopkins School of Public Health, Baltimore, MD United States; 11 Department of Medicine, Division of Infectious Diseases, Johns Hopkins School of Medicine, Baltimore, MD United States; 12 Department of Pathology, Johns Hopkins School of Medicine, Baltimore, MD United States; 13 Department of Radiology and Radiological Science, Johns Hopkins School of Medicine, Baltimore, MD United States; 14 Department of Neurosurgery, Johns Hopkins School of Medicine, Baltimore, MD United States; 15 Department of Neurology, Johns Hopkins School of Medicine, Baltimore, MD United States; 16 Department of Neuroscience, Johns Hopkins School of Medicine, Baltimore, MD United States; 17 The McKusick-Nathans Institute of Genetic Medicine, Johns Hopkins School of Medicine, Baltimore, MD United States

**Keywords:** Clinical pharmacology, Medical genetics, Predictive markers, Schizophrenia

## Abstract

The L-type calcium channel gene, CACNA1C, is a validated risk gene for schizophrenia and the target of calcium channel blockers. Carriers of the risk-associated genotype (rs1006737 *A* allele) have increased frontal cortical activity during working memory and higher CACNA1C mRNA expression in the prefrontal cortex. The aim of this study was to determine how the brain-penetrant calcium channel blocker, nimodipine, changes brain activity during working memory and other cognitive and emotional processes. We conducted a double-blind randomized cross-over pharmacoMRI study of a single 60 mg dose of oral nimodipine solution and matching placebo in healthy men, prospectively genotyped for rs1006737. With performance unchanged, nimodipine significantly decreased frontal cortical activity by 39.1% and parietal cortical activity by 42.8% during the N-back task (2-back > 0-back contrast; *P*_FWE_ < 0.05; *n* = 28). Higher peripheral nimodipine concentrations were correlated with a greater decrease in activation in the frontal cortex. Carriers of the risk-associated allele, *A* (*n* = 14), had a greater decrease in frontal cortical activation during working memory compared to non-risk allele carriers. No differences in brain activation were found between nimodipine and placebo for other tasks. Future studies should be conducted to test if the decreased cortical brain activity after nimodipine is associated with improved working memory performance in patients with schizophrenia, particularly those who carry the risk-associated genotype. Furthermore, changes in cortical activity during working memory may be a useful biomarker in future trials of L-type calcium channel blockers.

## Introduction

Cognitive deficits are a core symptom of schizophrenia, but currently available antipsychotic drugs improve mainly positive symptoms (e.g. hallucinations and delusions), leaving patients with often debilitating cognitive dysfunction and negative symptoms (e.g. apathy, avolition, anhedonia). Efforts to develop cognitive-enhancing agents have been hindered, in part, by a lack of understanding of the neural systems underlying the cognitive deficits in schizophrenia, and therefore a lack of plausible drug targets for this clinical domain.

The CACNA1C gene (alpha 1C subunit of the L-type voltage-gated calcium channel) has been identified by several independent studies as a genome-wide associated risk gene for both schizophrenia^1–4^ and bipolar disorder^5,6^. We previously reported that healthy adults who carry the CACNA1C SNP rs1006737 *A* allele, which is associated with risk for schizophrenia and bipolar disorder, had increased activation in the prefrontal cortex during the N-back working memory task, compared to non-risk genotype carriers^1^. Increased activation of the prefrontal cortex during working memory without change in performance on the task (i.e. inefficient cortical engagement) has been previously reported in patients with schizophrenia and their healthy siblings (who are at increased genetic risk for schizophrenia, but without illness)^7^. We also found that mRNA expression of CACNA1C is increased in the dorsolateral prefrontal cortex in human post-mortem brain samples that carry the risk-associated genotype^1^. Yoshimizu and colleagues found that increased CACNA1C mRNA expression in carriers of the risk-associated genotype results in increased L-type voltage-gated calcium channel current density in induced human neurons^6^. These data suggest that blocking the L-type calcium channel may improve cortical function in patients with schizophrenia.

The CACNA1C gene codes for the alpha 1C subunit of Cav1.2 channels and is the binding site for L-type calcium channel (LTCC) antagonists. Recently, Hayes et al. found that there were lower rates of psychiatric hospitalization and self-harm in patients with schizophrenia, bipolar disorder, and nonaffective psychosis who were taking LTCC antagonists^8^. Nimodipine is a dihydropyridine LTCC antagonist used to treat cerebral vasospasm after subarachnoid hemorrhage and has been shown to be effective in treating mood symptoms in case reports^9,10^ and treatment trials^11–14^ of bipolar and unipolar depression. To date, there have been no clinical trials using nimodipine to treat schizophrenia. Nimodipine is highly lipophilic and readily crosses the blood-brain barrier^15–17^, making it an ideal candidate to probe the effects of L-type calcium channel blockade on cortical activation during working memory.

We conducted the following pharmacoMRI study to evaluate the effects of nimodipine on brain activation during cognitive and emotional tasks in healthy subjects. Based on our previous imaging genetics study, we hypothesized that nimodipine would decrease frontal cortical activation during working memory in the context of fixed performance, and that this improvement in cortical physiological efficiency would be greatest in subjects carrying the CACNA1C risk-associated genotype.

## Methods and materials

### Subjects

All subjects provided written informed consent approved by the Johns Hopkins Institutional Review Board prior to study participation. Subjects were healthy, non-smoking, Caucasian, right-handed men 18–35 years old. Subjects were screened for current and past psychiatric illness using the Structural Clinical Interview for Diagnosis (SCID) of DSM-IV Disorders^18^. Subjects completed a history and physical examination, biochemical and hematological laboratory screens, MRI safety screen, and screened for drugs of abuse including alcohol. Subjects were nonsmoking, as confirmed with urine nicotine and cotinine screening. Subjects were excluded for past or current neurological or psychiatric illness, uncontrolled medical disorder, hypotension or uncontrolled hypertension, or head trauma with loss of consciousness or evidence of functional impairment after head trauma. Subjects did not take psychotropic medications within 3 months of the first study visit or any enzyme-inducing or inhibiting agent within 1 month of the study. All subjects were right-handed as determined by the Edinburgh Inventory^19^. A blood sample was collected and processed using standardized methods to extract DNA. Targeted genotyping of rs1006737 was done using a standard allelic discrimination Taqman assay, as described in the supplement.

### Study Design

The study was a randomized, double-blind, cross-over of a single 60 mg dose of oral nimodipine (20 mL of Nymalize®) and a matching placebo solution. An investigational pharmacist randomized subjects to receive either nimodipine or placebo for the first visit followed by the opposite treatment on the second visit. Visits were separated by a washout period (minimum 2-weeks). *Nymalize* and matching placebo were donated by Arbor Pharmaceuticals, LLC. Subjects were screened for drugs of abuse on the morning of each study visit and an intravenous catheter was placed in their left forearm for collection of multiple blood samples. Subjects completed the Profile of Mood States (POMS) at the screening visit and then upon study visit admission and 15, 90, and 240 min after drug administration on each study visit to assess subjects’ mood during the study and compare between treatments.

### Nimodipine pharmacokinetics

Blood samples (10 mL) were taken just before and approximately 15, 30, 40, 50, 60, 70, 80, 90, 240 min after drug/placebo administration. Samples between 40 and 90 min were taken in between each of the fMRI tasks. Samples were processed using centrifugation to separate plasma, using standard techniques. Nimodipine was extracted from plasma via protein precipitation, and drug quantification was performed using a validated liquid chromatographic-mass spectrometric (LC-MS) method, with a lower limit of quantification of 0.2 ng/mL. Full method details are described in the supplement.

### MRI acquisition

The first 15 subjects were scanned on the Siemens 3T Trio MRI and the last 15 subjects were scanned on the same magnet upgraded to the Siemens 3T Prisma MRI. All scanning parameters remained the same between the Trio and Prisma acquisitions. A structural MRI (T1-weighted 3D MPRAGE) was completed on each subject and reviewed by a neuroradiologist to ensure there were no structural abnormalities. The first functional MRI task was started ~30 min following drug/placebo administration. Subjects were trained or instructed on the fMRI tasks prior to scanning. Subjects completed the N-back working memory task^1,7^, a modified monetary incentive delay task^20^, an emotional memory task^1^, an emotional face matching task^21^, and the flanker task^22^, as previously described. For the functional MRI tasks, we acquired single-shot GRE echoplanar BOLD images using the Siemens 3D PACE (Prospective Acquisition CorrEction) MOCO (MOtion COrrection) sequence^23^. The PACE MOCO sequence, which prospectively corrects for motion during scan acquisition, eliminates subsequent need for realignment, thus minimizing the impact of motion on data quality.

### N-back working memory task

The N-back working memory task was scanned second in the series of fMRI tasks, ~45 min to an hour after nimodipine/placebo administration, which corresponds with peak nimodipine levels^17^. Neural activity was measured using blood oxygenation level dependent (BOLD) fMRI while subjects performed the N-back. Details of the N-back task have been previously described^1,7^. Briefly, there were 4 blocks of the 2-back (i.e. working memory condition) alternating with a 0-back (i.e. control condition). During the 0-back blocks, the subject serially responds with the current digit presented (1,2,3,4 in a diamond shaped box). During the 2-back block, the subject responds with number presented 2 previous (“*N*” = 2). Subjects were trained on the N-back task until they could perform the 2-back condition above 80% accuracy twice in a row. BOLD fMRI parameters for the N-back task were the following: axial slices = 33, thickness = 3.0 mm, voxel size=3.8 × 3.8 × 3.0 mm, TR = 2000 ms, TE = 28 msec, FOV = 240 mm, matrix = 64 × 64.

### Functional imaging analyses

Functional MR images were processed and analyzed using Statistical Parametric Mapping (SPM12) (http://www.fil.ion.ucl.ac.uk/spm) implemented in MATLAB (Mathworks Inc., Natick, MA, USA). (http://www.fil.ion.ucl.ac.uk/spm). For each task, functional scans (*n* = 124 for the N-back task) were acquired for each participant to measure the T2*-weighted BOLD signal. Dummy scans (*n* = 2–3) were acquired at the beginning to allow for steady-state magnetization and discarded from subsequent analyses. Prospective motion-corrected images were spatially normalized to the first EPI scan, smoothed with a 10-mm full-width half-maximum (FWHM) Gaussian filter, and ratio normalized to the whole-brain global mean.

In the first-level analyses, for each task, linear contrasts were computed producing t-statistical parameter maps at each voxel. For example, t-statistical parameter maps were produced for the 2-back condition greater than 0-back condition contrast to isolate brain regions related to working memory. These statistical images were entered in a second-level model to identify significant activations within and between treatments and genotype groups.

The main effect of nimodipine on brain activity related to working memory (2-back > 0-back) was analyzed using a paired *t*-test (placebo versus nimodipine) and the contrast was thresholded at pFWE < 0.05 and 10 contiguous voxels (spatial extent threshold). To determine the effect of rs1006737 genotype on the main effect of nimodipine, the Image Calculator (ImCalc facility) in SPM12 was used to create images of Placebo-Nimodipine for each subject. These images were then entered into a multiple linear regression (an additive genetic model using copies of *A* allele), and masked for the main effect of nimodipine on N-back task activation. The interaction of genotype*nimodipine was thresholded at *p* < 0.001 uncorrected and extent *k* = 10 voxels. First and second-level analyses were conducted for the other fMRI tasks, as previously described^1,20–22^.

We conducted exploratory analyses to determine if there was a relationship between nimodipine concentrations, diastolic or systolic blood pressure, or heart rate, and the main effect of nimodipine on N-back working memory-related neural activation. A multiple linear regression was done for each covariate (regressed on images of Placebo-Nimodipine using ImCalc), masked for the main effect of nimodipine on N-back task activation, and thresholded at *p* < 0.001 uncorrected and extent *k* = 10 voxels for each analysis.

### Physiological measurements

During fMRI scanning, a pulse oximeter was placed on their left middle finger and measured heart rate through a physiological signals acquisition unit (MP150, BioPac Systems Inc., Santa Barbara, CA) equipped with AcqKnowledge 3.7 software. Additionally, vital signs (heart rate and blood pressure) were measured outside of the MRI at baseline (study visit admission), just before the administration of nimodipine/placebo (pre-dose), 30 min after dosing (pre-MRI), just after the MRI, 1 h and 2 h after the MRI, and before discharge around 4 h after drug administration. Blood pressures and heart rates were compared between placebo and nimodipine treatments using paired t-tests. A Bonferroni corrected p-value of 0.01 was considered statistically different between treatments.

### Cognitive performance data

Performance on the N-back task was assessed through a fiber optic response box, which recorded both percent correct (accuracy) and reaction time (RT). Reaction times faster than 100 milliseconds (ms) or slower than 1700 ms (300 ms before stimuli) were removed from the analyses to eliminate anticipatory responses to the stimuli. A paired t-test was used to compare accuracy and reaction times between conditions (0-back and 2-back) and between treatments (nimodipine and placebo) using GraphPad Prism 8, for Windows, GraphPad Software, La Jolla California USA.

### Mood assessment

Subjects completed the POMS at the screening visit, upon admission, and 15, 90, and 240 min after drug administration on each study visit to assess subjects’ mood during the study and compare between treatments, as described in more detail in the supplement.

## Results

Thirty subjects completed the study. Subjects were between 19 and 33 years old (mean [SD] age, 25.8 [3.68]). One subject was missing from the N-back fMRI analyses because the response box failed during one visit. One subject was removed from the N-back fMRI analyses because he performed below 70% correct on the 2-back on both study visits. A total of 28 subjects were included in the N-back fMRI analyses, who performed > =75% correct for the 2-back condition on both study visits. Of the 28 subjects, 14 were carriers of rs1006737 GG, 4 carried GA, and 10 carried the AA risk-associated genotype.

### Effect of nimodipine on neural activation

**Fig. 1:**
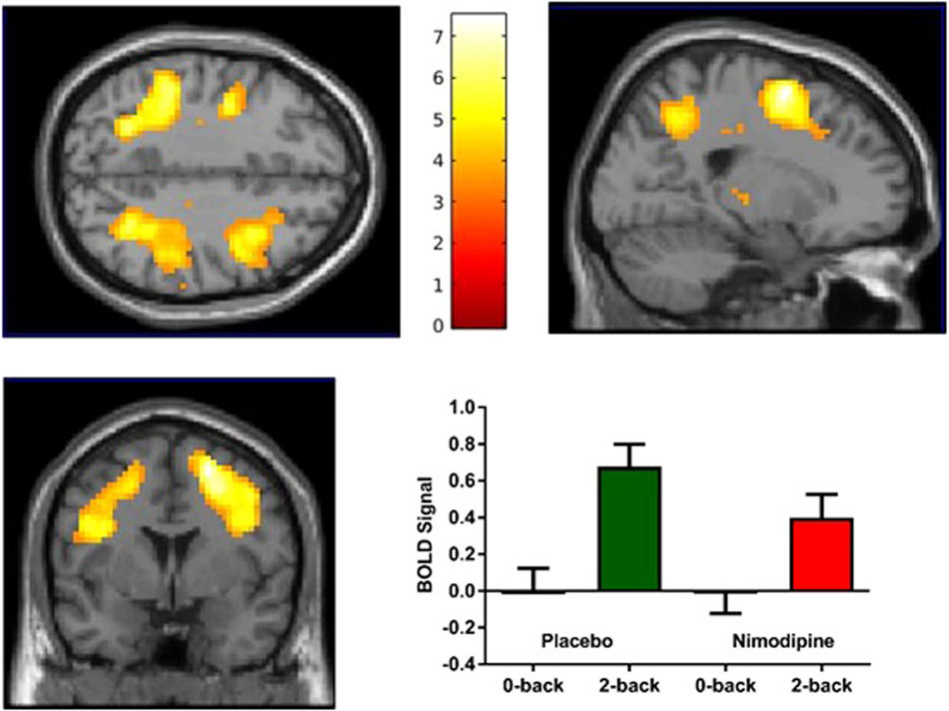
Nimodipine decreases frontal and parietal cortical activity during working memory (group-averaged map, threshold *p* < 0.001 uncorrected for visualization purposes). A. Axial view. B. Sagittal view. C. Coronal view. D. BOLD signal in peak voxel [21,2,59] is being driven by a decrease in activation during 2-back (working memory contrast).

Nimodipine significantly decreased cortical brain activity in the frontal and parietal cortices during the N-back working memory task (Fig. 1). Table 1 shows the clusters that are significantly decreased after nimodipine compared to placebo (threshold *p*_FWE_ < 0.05 and *k* = 10). There were no brain regions that were significantly increased after nimodipine compared with placebo, even at a lower statistical threshold (*p* < 0.001 uncorrected, *k* = 10). Because the MRI system was upgraded halfway through the study, we also analyzed the N-back fMRI data for the first half and the second half separately, which resulted in similar effects in the frontal and parietal cortical regions. There was no order effect, as we found no significant differences between visit 1 and visit 2. There were no significant differences in neural activation between nimodipine and placebo treatments during any of the other fMRI tasks, including: the modified monetary incentive delay task, which activates the striatum during motivation^20^; an emotional memory task, which activates the prefrontal cortex, amygdala, and hippocampus during encoding and retrieval of aversive scenes^1^; an emotional face matching task, which activates the amygdala^21^; and the flanker cognitive control task, which activates the anterior cingulate cortex during response inhibition^22^.

**Table 1:** Brain regions with significantly decreased neural activity during working memory after nimodipine.

pFWE peak voxel	*T*	*Z*	Voxels	Coordinates	Brain region
0.001	7.48	5.46	88	21,2,59	Superior frontal cortex
0.001	7.29	5.37	24	−42,−1,29	Precentral gyrus
0.001	7.28	5.37	31	−24,−55,41	Superior parietal cortex
0.005	6.69	5.09	37	−48,−37,44	Inferior parietal cortex
0.022	6.01	4.75	16	30,−58,35	Superior parietal cortex

### Nimodipine effect is concentration-dependent in the frontal cortex

**Fig. 2:**
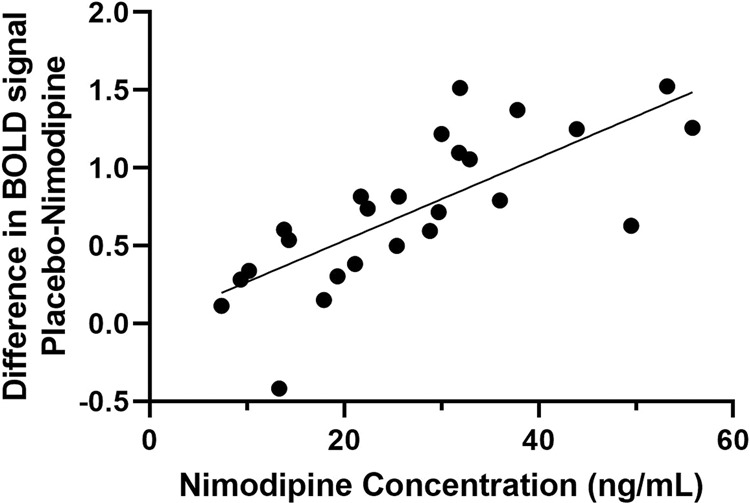
Higher nimodipine concentrations (measured at the time of N-back task) are associated with a greater decrease in frontal cortical activation during working memory (peak voxel [54,17,−4]).

Plasma nimodipine concentrations were significantly correlated with a greater decrease in neural activity after nimodipine (i.e. difference between placebo and nimodipine) in the inferior frontal cortex (peak voxel [54,17,−4], *T* = 5.37; linear regression with betas from peak: *p* = 1.87e-06, *r*^2^ = 0.56, Fig. 2), with higher concentrations resulting in a greater decrease in frontal activation. Other regions where higher concentrations were associated with a greater decrease in neural activity, including the parietal cortex, can be found in Table 2. There were no brain regions where nimodipine concentrations were significantly inversely correlated with a decrease in brain activation after nimodipine compared with placebo (threshold *p* < 0.001 uncorrected, 10 voxels). There were also no brain regions where higher nimodipine concentrations were significantly correlated with increased neural activity. Concentrations were not different between CACNA1C genotype groups. Supplementary Fig. 1 shows individual nimodipine concentration-time profiles.

**Table 2:** Higher nimodipine concentrations were associated with a greater decrease in neural activity after nimodipine.

*T*	Z	Voxels	Coordinates	Brain region
5.37	4.28	24	54,17,−4	Inferior frontal cortex
4.84	3.98	37	−30,−61,65	Superior parietal cortex
4.59	3.83	15	27,−73,53	Superior parietal cortex
4.54	3.80	39	45,−40,50	Supramarginal gyrus
4.42	3.72	11	−48,−49,50	Supramarginal gyrus
4.24	3.61	70	3,20,38	Prefrontal cortex (SMA)
4.10	3.51	16	15,−61,62	Superior parietal cortex

### Interaction of CACNA1C genotype with nimodipine effect on neural activation

**Fig. 3:**
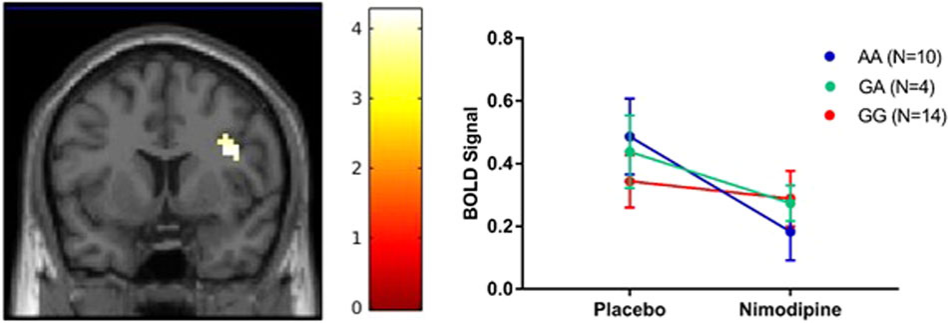
Carriers of the CACNA1C risk-associated allele (*A*) had a greater decrease in neural activation after nimodipine than non-risk allele carriers (*GG*). Panel A shows brain activity that decreased more in rs1006737 A allele carriers than GG carriers. Panel B shows BOLD signal change in the peak voxel [39,8,23].

Carriers of the risk-associated allele, *A*, had a greater decrease in frontal cortical activation during working memory compared to non-risk allele carriers (linear regression thresholded at *p* < 0.001 uncorrected, *k* = 27; peak voxel: *p*_FWE_ = 0.046, *T* = 4.24, *Z* = 3.66, [39,8,23]; Fig. 3). While the effect of the risk-allele only trends towards significance after FWE correction, the interaction of genotype × drug is located in the peak cluster for the main effect of nimodipine on working memory. Subjects who carried the AA genotype had a 62.32% decrease in BOLD activation after nimodipine compared to 37.42% for GA carriers and 16.05% for GG carriers (Fig. 3). There were no brain regions where *G* allele carriers had a significantly greater decrease than *A* allele carriers during working memory (threshold *p* < 0.001 uncorrected, 10 voxels).

### Working memory performance

Subjects had higher accuracy on the 0-back (>99%) compared to the 2-back for both placebo and nimodipine treatments (*p* < 0.0001). Performance was not significantly different on the 2-back between placebo (91.6%, SD = 8.07) and nimodipine (91.5%, SD = 8.48) treatments (*p* = 0.90). Accuracy was not significantly different between genotype groups for any condition. Accuracy on the 2-back was not correlated with BOLD signal in the peak voxel [21,2,59] of the main effect of nimodipine.

Subjects responded faster during the 2-back compared to the 0-back (241 ms, SD = 0.102, vs. 401 ms, SD = 0.060) (241 [0.102] ms, vs. 401 [0.060] ms for nimodipine), likely because they were primed for the button press for the 2-back since they knew the number they would be pressing in advance. Reaction time was not significantly different between nimodipine and placebo treatments or between genotype groups for either condition (*p* > 0.05). Reaction time on the 2-back was not correlated with BOLD signal in the peak voxel [21,2,59] of the main effect of nimodipine.

### Physiological measurements

In general, peripheral blood pressure remained stable across the study visits (Supplementary Fig. 2). Diastolic blood pressure was 13.6%, SD = 8.901, lower 30 min after nimodipine administration (pre-MRI) as compared with placebo (paired *t*-test: *t* = 6.297, df = 29, *p* = 7.08e-07), but was not significantly different at other time points. Systolic blood pressure was not significantly different between treatments at any time point. Changes in blood pressure were not different between genotype groups. Neural activity (BOLD signal) in the peak voxel of the main effect of nimodipine was not correlated with diastolic or systolic blood pressure.

Average heart rate increased 15.6%, SD = 10.5, 30 min after nimodipine administration, which corresponds with the decrease in blood pressure (Supplementary Fig. 3; *p* < 0.0001). During the N-back task, heart rate was continuously higher after nimodipine compared with placebo (repeated-measures two-way ANOVA across all blocks *F*(1,22) = 6.458, *p* = 0.019, SD = 6.028; Supplementary Fig. 3). Heart rate was significantly higher during 2-back as compared to the 0-back during both placebo (paired t-test *p* = 0.0068) and nimodipine (paired *t*-test *p* = 0.0003) treatments. Heart rate was not correlated with BOLD signal in the peak voxel of the main effect of nimodipine. Change in heart rate was similar between genotype groups.

### Mood states

There were no differences between nimodipine and placebo visits (at any time point) for any of the mood states, domain scores, or for the total mood disturbance score. Subjects reported not being able to tell which visit they received nimodipine.

## Discussion

In healthy subjects, nimodipine administration significantly decreased neural activity in parietal and frontal cortices during the N-back working memory task, despite fixed performance on the task. This is a pattern of change that suggests that nimodipine improves the physiological efficiency of cortical processing of working memory information. There were no effects of blood pressure or heart rate on our pharmacoMRI results, supporting the notion that the present drug and genotype effects on BOLD signal are indicative of a central nervous system effect. The effect of nimodipine appears to be relatively selective to working memory, as it did not affect neural activation during encoding or retrieval of aversive or neutral scenes, emotional face matching, attention, and cognitive control, or motivation and/or reward tasks. The apparent specificity of nimodipine’s effects on the working memory circuitry further suggests the changes are not related to global alterations in peripheral blood pressure or brain perfusion. The effects of nimodipine on frontal and parietal cortical activity during working memory were concentration-dependent, with higher concentrations resulting in lower frontal cortical activity. There were also several regions of the parietal cortex and SMA that showed a similar relationship, where higher concentrations had a greater decrease in neural activity. The larger reduction in frontal–parietal network activity, involving the parietal cortex (superior parietal, supramarginal gyrus) as well frontal areas (including SMA), may reflect the network involved in the representation of quantity (numbers) during working memory with a button press motor response. The N-back task has been shown to have high within-subject and group-level reliability, measured by a high intraclass correlation coefficient with retest scans, which makes this paradigm ideal for pharmacoMRI studies^24^.

Carriers of the CACNA1C risk allele (*A*) had a greater decrease in frontal cortical activity after nimodipine, providing further evidence this effect is being driven by changes in calcium channel activity. We previously showed that CACNA1C risk allele carriers have increased CACNA1C expression in the dorsolateral PFC and hyperactivation of the prefrontal cortex during working memory^1^. If risk allele carriers have greater L-type calcium channel expression in the prefrontal cortex, the specific target of nimodipine, it may not be surprising that they would show the greatest effect in this region. However, evidence that CACNA1C risk-associated genotype predicts expression of this channel subunit is inconsistent^25^. Since all genotype groups had a decrease in prefrontal cortical activity after nimodipine, and there was no difference in nimodipine’s effect in the parietal cortex between genotype groups, we would predict that nimodipine could improve performance in all patients who have a working memory deficit, not just those carrying the risk genotype.

Nimodipine administration did not improve performance on the N-back task in these healthy subjects without a working memory deficit, which was to be expected since the N-back test is not designed to be behaviorally sensitive to change, particularly in subjects who can easily perform this task well. The subjects performed near or at a ceiling during both placebo and nimodipine (91% mean accuracy during the 2-back condition) and subjects were trained to perform at or above 80% accuracy. Our principal aim in this study was to assess the effect of nimodipine on cortical efficiency, a phenomenon associated with increased risk for schizophrenia, which is defined operationally as a change in physiological activity without a change in performance. Nimodipine has shown pro-cognitive effects in several animal models of cognitive impairment^26–31^, including improved spatial working memory performance in both young and aged rats^32–35^, and improved associative learning in aging rabbits^30^. Nimodipine also improved performance on a delayed-response memory task in aged nonhuman primates^36^. Other animal studies have found that excessive calcium signaling can impair prefrontal cortical functioning, which then requires more extensive activation to compensate^35,36^, which may explain the pro-cognitive effects of inhibiting calcium channel signaling. Research in nonhuman primates has shown that calcium actions in the prefrontal cortex can impair the information held in working memory through either neuronal hyperexcitability or calcium-cAMP signaling-dependent opening of potassium channels^37,38^. In humans, chronic treatment with nimodipine (30 mg TID for 12 weeks) resulted in better cognitive performance in patients with dementia and cognitive impairment in an open-label trial^39^ and randomized, placebo-controlled, double-blind trials^40–42^. Studies are needed to test the effects of nimodipine on working memory performance and other cognitive domains in patients with schizophrenia and related disorders, who have cognitive deficits and therefore do not perform as well on the N-back task. Future studies should test multiple doses and cognitive testing outside of the fMRI to determine the dose-response relationship and determine nimodipine’s effects on other cognitive domains. Clinical studies of the cognitive effects of nimodipine should include functional MRI as a biomarker of treatment response, which would also provide insight into the mechanism behind nimodipine’s pro-cognitive effects.

## Supplementary information

Supplemental methods and results
