# Supplementary material for: Nimodipine improves cortical efficiency during working memory in healthy subjects
Source: Transl Psychiatry. 2020 Nov 2;10:372. doi: 10.1038/s41398-020-01066-z (PMC7606375; doi:10.1038/s41398-020-01066-z)

**Supplemental Methods**

**Genotyping**

Our goal was to recruit 30 subjects; at least 10 subjects who carried AA genotype and at least 10 who carried the GG genotype. DNA was extracted by performing cell lysis on 10mL of whole blood using Qiagen RBC Lysis Solution and Cell Lysis Solution. Protein and DNA were precipitated from lysed cells using Protein Precipitation solution and 2-propanol. Quality control of DNA concentrations was determined using Qubit dsDNA High Sensitivity Assay Kits. The CACNA1C single nucleotide polymorphism rs1006737 was determined by standard allelic discrimination Taqman assay that uses the 5′ nuclease activity of Taq DNA polymerase to detect a fluorescent reporter signal generated after PCR amplification. The assay cocktail (Assays on Demand) for rs1006737 was obtained from Applied Biosystems (Foster City, CA). Genotype reproducibility was routinely assessed by re-genotyping all samples for the selected SNP and was 100%.

**Nimodipine assay**

Samples were prepared for analysis via protein precipitation and evaporated to dryness, followed by reconstitution in a 1:1 ratio of 0.1% formic acid in water (mobile phase A) to 0.1% formic acid in acetonitrile (mobile phase B). Chromatographic separation was achieved using a Kinetex C18 column, 2.6 µm 50 x 2.1 mm (Phenomenex, Torrance, CA) on an Acquity LC system (Waters Corporation, Milford, MA). Nimodopine, as well as the deuterated internal standard, nimodipine-d7, were monitored on a QTRAP 5500 mass spectrometer (SCIEX, Foster City, CA). Nimodipine and nimodipine-d7 transitions used for quantification were *m/z* 419.44→343.10 and *m/z* 426.48→350.40, respectively. Total analytical run time was 2.0 min, and the analytical measuring range of the assay for nimodipine quantification was 0.2 to 2000 ng/mL. The assay was validated in accordance with FDA Guidance for Industry: Bioanalytical Method Validation recommendations, and all results met acceptability criteria. The inter- and intra-assay precision and accuracy values were within the ±20% acceptance criteria with precision values ≤13.1% and accuracy values ranging between -14.5% and 11.0% for the quality control levels, including the lower limit of quantification (LLOQ).

**Psychological rating scale**

The Profile of Mood States (POMS short form) was used to assess whether nimodipine altered subjects’ moods. The self-report rating scale is used to capture transient and fluctuating feelings or moods and consists of 37 items that can be combined for a total mood disturbance score, and six mood clusters: anger-hostility, confusion-bewilderment, depression-dejection, fatigue-inertia, tension-anxiety, and vigor-activity. Each mood is rated on a scale of 1 to 5 (1=not at all and 5=extremely). The POMS was administered before and 15, 90, and 240 minutes after drug/placebo administration.

**Supplemental Results**

**Supplmental Figure 1.** Individual nimodipine concentration-time profiles after a single 60 mg dose of oral Nymalize® solution administered at time 0 hours.
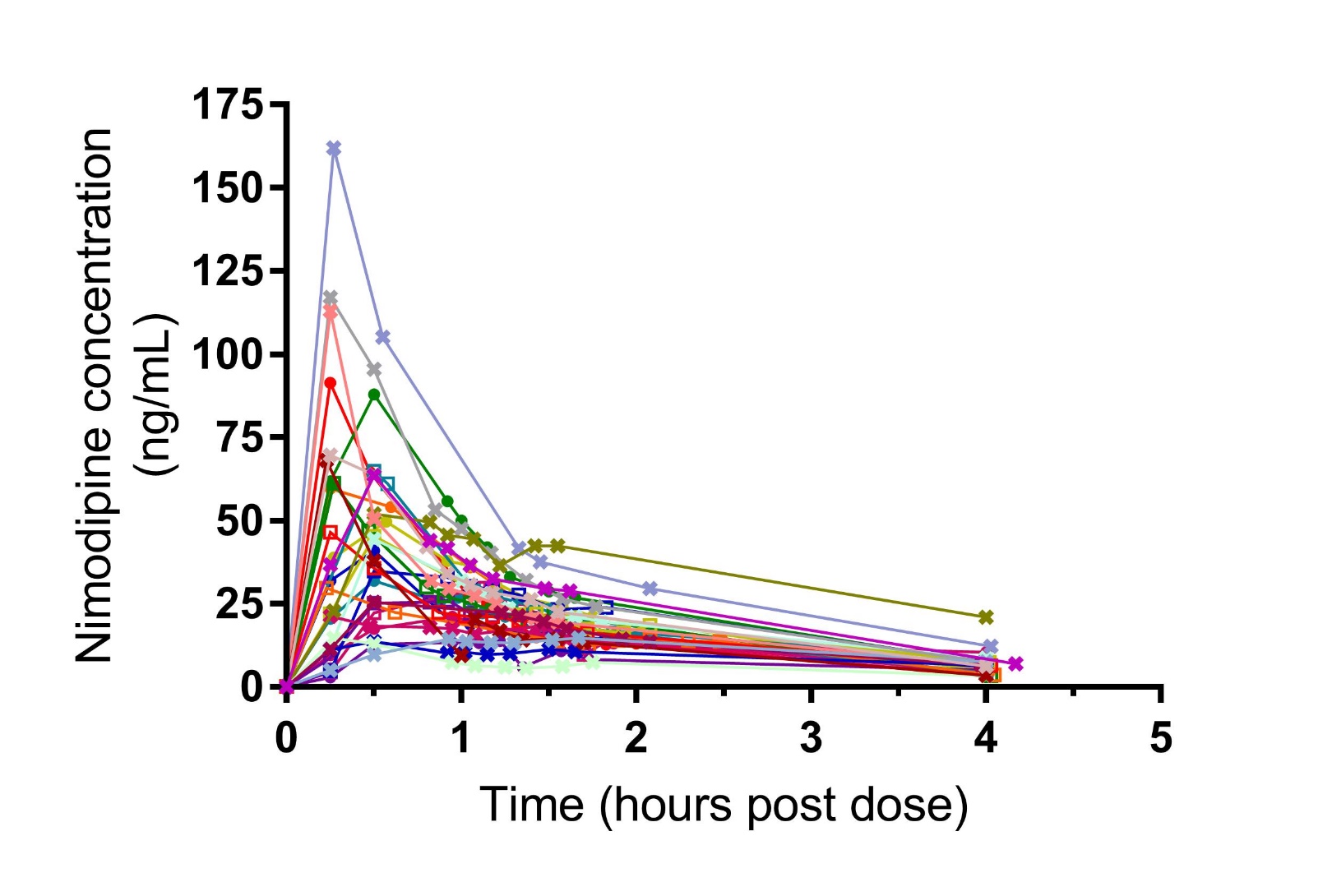


**Supplemental Figure 2.** Nimodipine transiently reduced diastolic blood pressure approximately 30 minutes after administration (paired t-test: t=6.297, df=29, p=7.08e-07, SD= 8.901), but was not significantly different at other time points. Systolic blood pressure was not significantly different between treatments at any time point.


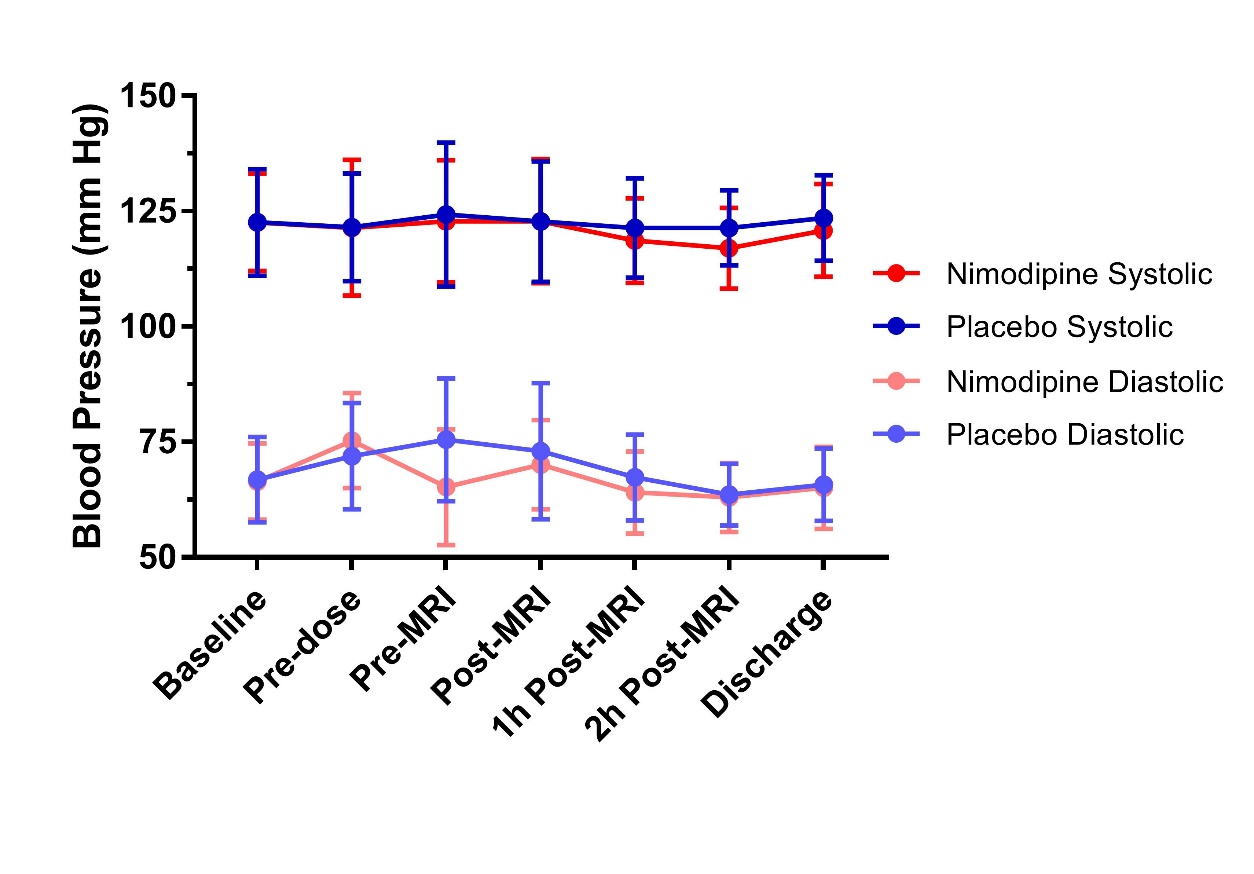


**Supplemental Figure 3.** Heart rate was significantly greater during nimodipine treatment than during placebo treatment during the N-back task (p<0.019 for repeated measures ANOVA). The first stimulus block (0-back) begins at time 0 sec and ends at the first error bar, followed by the second stimulus block (2-back) ending at the second error bar, and continue alternating between 0-back and 2-back blocks.


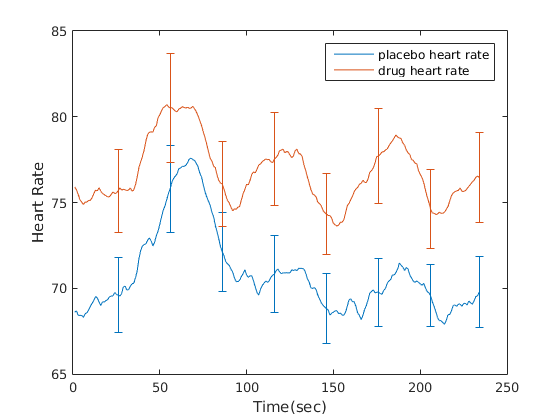

Supplement: Supplementary file 1 — Supplemental methods and results [file 41398_2020_1066_MOESM1_ESM.docx]
